# Calycosin inhibited autophagy and oxidative stress in chronic kidney disease skeletal muscle atrophy by regulating AMPK/SKP2/CARM1 signalling pathway

**DOI:** 10.1111/jcmm.15514

**Published:** 2020-09-10

**Authors:** Rong Hu, Ming‐qing Wang, Ling‐yu Liu, Hai‐yan You, Xiao‐hui Wu, Yang‐yang Liu, Yan‐jing Wang, Lu Lu, Wei Xiao, Lian‐bo Wei

**Affiliations:** ^1^ School of Traditional Chinese Medicine Southern Medical University Guangzhou China; ^2^ Shenzhen Hospital Southern Medical University Shenzhen China; ^3^ Zhongshan Huangpu People's Hospital Zhongshan China; ^4^ Zhujiang Hospital of Southern Medical University Guangzhou China; ^5^ The First Affiliated Hospital Guangzhou University of Chinese Medicine Guangzhou China

**Keywords:** AMPK, autophagy, Calycosin, CARM1, oxidative stress, skeletal muscle atrophy, SKP2

## Abstract

Skeletal muscle atrophy is a common and serious complication of chronic kidney disease (CKD). Oxidative stress and autophagy are the primary molecular mechanisms involved in muscle atrophy. Calycosin, a major component of *Radix astragali*, exerts anti‐inflammatory, anti‐oxidative stress and anti‐autophagy effects. We investigated the effects and mechanisms of calycosin on skeletal muscle atrophy in vivo and in vitro. 5/6 nephrectomy (5/6 Nx) rats were used as a model of CKD. We evaluated bodyweight and levels of serum creatinine (SCr), blood urea nitrogen (BUN) and serum albumin (Alb). H&E staining, cell apoptosis, oxidative stress biomarkers, autophagosome and LC3A/B levels were performed and evaluated in skeletal muscle of CKD rat. Calycosin treatment improved bodyweight and renal function, alleviated muscle atrophy (decreased the levels of MuRF1 and MAFbx), increased superoxide dismutase (SOD), catalase (CAT), and glutathione peroxidase (GSH‐Px) activity and reduced malondialdehyde (MDA) levels in skeletal muscle of CKD rats. Importantly, calycosin reduced autophagosome formation, down‐regulated the expression of LC3A/B and ATG7 through inhibition of AMPK and FOXO3a, and increased SKP2, which resulted in decreased expression of CARM1, H3R17me2a. Similar results were observed in C2C12 cells treated with TNF‐α and calycosin. Our findings showed that calycosin inhibited oxidative stress and autophagy in CKD induced skeletal muscle atrophy and in TNF‐α‐induced C2C12 myotube atrophy, partially by regulating the AMPK/SKP2/CARM1 signalling pathway.

## 1 INTRODUCTION

Chronic kidney disease (CKD) is a global public health problem that affects 10%‐15% of the worldwide adult population.^1^ Chronic kidney disease results in reduced life quality, and increased risk of diverse complications such as cardiovascular disease, diabetes, infection, cancer and muscle atrophy, which can further cause high morbidity and mortality of CKD.^2^, ^3^ Skeletal muscle wasting is characterized by reduced muscle fibre cross‐sectional area and protein content, and loss of muscle mass, strength and function.^4^ Malnutrition has been historically believed to be responsible for protein loss in the CKD patients. However, increased dietary protein has not been shown to reverse protein loss in CKD models experiments.^3^ Muscle mass is maintained by balancing protein metabolism between protein biosynthesis and degradation.^5^ Studies have shown that the pathophysiological mechanisms and risk factors for CKD‐induced muscle atrophy include metabolic acidosis, inflammation, changes in levels of angiotensin II, vitamin D deficiency, increased oxidative stress and over‐autophagy. These changes have been shown to promote muscle protein loss through increased protein degradation and decreased protein biosynthesis, and impaired muscle growth and repair.^6^, ^7^ Current treatments for CKD‐induced muscle atrophy include nutritional supplementation, reduction of insulin resistance, androgenic steroids, neuromuscular electrical stimulation, aerobic exercise and muscle training. However, more effective treatment strategies for CKD‐induced muscle atrophy are needed.

Oxidative stress and autophagy are the major mechanisms of skeletal muscle atrophy.^8^, ^9^, ^10^ Studies have shown that high level reactive oxygen species (ROS) production can induce mitochondrial dysfunction and activate forkhead box class O (FOXO) transcription factors in skeletal muscles, resulting in muscle atrophy.^11^, ^12^ Appropriate levels of ROS are produced by each muscle fibre during its contraction and relaxation and are maintained a balance between pro‐oxidant and antioxidant molecules, which contributes to development and regeneration of skeletal muscles.^13^, ^14^ However, under pathological conditions, ROS can increase in skeletal muscles, which results in disruption of cellular redox status and subsequent damage to cellular biomolecules, including lysosome‐mediated autophagy, the ubiquitin‐proteasome system (UPS) and the caspase‐3 proteolytic pathway, leading to reduced muscle through enhanced degradation of muscle proteins.^13^

Autophagy, characterized by lysosome‐dependent turnover of cellular organelles and proteins, is an important process in maintenance of muscle mass. Muscles with impaired autophagy subjected to fasting or denervation exhibit a severe atrophic phenotype.^15^ In contrast, excess autophagy can aggravate cellular damage by engulfing proteins, mitochondria, and other organelles that are necessary for normal cellular function.^16^ Autophagy has been shown to play a crucial role in muscle atrophy. A recent study showed patients with CKD exhibited increased skeletal muscle atrophy, autophagy and mitophagy, and increased Foxo3 activation.^17^ However, further studies are needed to characterize molecular regulation of autophagy and oxidative stress in CKD‐induced skeletal muscle atrophy.

Studies have found that the AMPK–SKP2‐CARM1 signalling pathway is a new signal transduction axis in the regulation of autophagy induction under the induction of nutritional starvation. Co‐activator associated arginine methyltransferase 1 (CARM1) as an important component of mammalian autophagy, while histone arginine methylation that relies on CARM1 is Key nuclear events in autophagy. Under starvation because of nutritional deficiencies, AMP‐activated protein kinase (AMPK) phosphorylation results in the activation of FOXO3a transcription in the nucleus, which in turn inhibits SKP2, and this inhibition leads to an increase in CARM1 protein levels and subsequent increase in histones H3 Arg17 dimethylation levels, activate transcriptional expression of downstream autophagy genes.^18^ Other studies have also shown that the lack of nutrients will increase the AMP: ATP ratio, which will lead to AMPK activation and phosphorylation of TSC2 and RPTOR to inhibit MTORC1. In addition, AMPK can activate autophagy by directly phosphorylating ULK1/2, and participate in bone Muscle atrophy.^10^ Another study found that CARM1 may be a new target for muscle wasting. The increase of CARM1 protein level is positively related to the loss of muscle mass during denervation in mice. Knocking down CARM1 expression in vivo or in vitro can inhibit the expression of muscle atrophy‐related genes Atrogin‐1 and MuRF1, and slow the process of muscle atrophy. The underlying mechanism may be that CARM1 interacts with FoxO3, and FoxO3‐dependent transcription requires methylation modification through CARM1.^19^ However, the mechanism of AMPK/ SKP2/CARM1 in skeletal muscle atrophy is unknown. The purpose of this study was to investigate the mechanism of CKD‐induced skeletal muscle atrophy based on AMPK/ SKP2/ CARM1 signalling pathway and find effective treatments or targets.

*Radix astragali* is a well‐known traditional Chinese herbal medicine that has been used for more than 2000 years. The dry root of *Radix astragali* contains multiple components such as calycosin, saponins, polysaccharides, isoflavonoids and astragalosides.^20^, ^21^ Calycosin (7,3′‐dihydroxy‐4′‐methoxyisoflavone (C_16_H_12_O_5_)), the main bioactive chemical in *Radix astragali*, exerts anti‐inflammatory, anti‐oxidative stress, antidiabetic, anti‐tumour and pro‐angiogenic effects.^22^ A recent study showed that calycosin inhibited cell proliferation, and induced cell death via caspase‐3‐dependent apoptosis and cytotoxic autophagy in HT29 CRC cells.^23^ However, the effects of calycosin on autophagy, oxidative stress, and cell apoptosis in muscle atrophy have not been characterized. In this study, we evaluated the effects of calycosin on skeletal muscle atrophy in vivo and in vitro.

## 2 MATERIAL AND METHODS

### 2.1 Animal experiments design

All animal experiments were approved by the Institutional Animal Care and Use Committee (IACUC) of Southern Medical University (Guangzhou, China). Eight‐week‐old male Sprague‐Dawley rats (180‐220 g) were purchased from the Experimental Animal Center of Southern Medical University. The rats were housed in a climate‐controlled with 12 hours day/night cycles and allowed to free food and water. The rats were randomly divided into a sham‐operation group (Sham, n = 10) and a 5/6 nephrectomy (5/6 Nx, n = 30) group. The CKD rats were subjected to 5/6 nephrectomy by removing the upper and lower thirds of the de‐capsulated left kidney, then subjected to right nephrectomy a week later, as described previously.^24^ The control (Sham) group underwent a sham operation. Bodyweight was measured every two weeks. Between 1 and 3 weeks after surgery, eight rats died. Levels of Scr, Bun, HB and Alb were measured 8 weeks after surgery. Rats that successfully underwent 5/6 nephrectomy were randomly assigned to two groups: the 5/6 Nx model group (n = 11) treated with saline water by gavage, and the calycosin‐treated group (n = 11), which was administered calycosin (15 mg/kg, Intraperitoneal injection) daily for 8 weeks. Following the treatment course, the rats were killed by abdominal main blood draw under general anaesthesia. The bodies were weighed and blood, kidney tissue, gastrocnemius (GAS), tibial anterior (TA) muscles were collected on ice. Blood was hold still for 30 minutes‐2 hours at and then centrifuged at 3000 g for 15 minutes to obtain the serum. The kidneys and muscles were cut into two portions. One portion was fixed in 4% paraformaldehyde for histological analysis, and the other portion was snap‐frozen in liquid nitrogen and stored at −80°C for protein analysis.

Calycosin was purchased from Solvay (Beijing) Co., Ltd., article number: SC8040; CAS number: 20575‐57‐9; molecular formula: C16H12O5; molecular weight: 284.26; purity: HPLC ≥ 98%. Based on previously references and the equivalent dose calculated for rats were 15 mg/ kg. 5 mL DMSO and 3 g Calycosin were made up to 6 mL of mother liquor and dilute with 15 mg/mL in physiological saline, stored in a dark place at 4°C.

Serum creatinine (Scr), blood urea nitrogen (BUN) and serum albumin (Alb) were quantified using anautomatic biochemical analyzer. Blood haemoglobin was determined using an Aim Strip Hb meter (Ermarine Laboratories Inc, San Antonio, TX, USA).

### 2.2 Cell culture

C2C12 cells were purchased from the Chinese Academy of Science Cell Bank (Shanghai, China). Cells were cultured in DMEM‐F12 (Gibco, USA) supplemented with 10% foetal bovine serum (FBS) (Gibco, BRL, USA), 100 units/ml of penicillin and 100 µg/mL of streptomycin (Gibco, New York, USA) in a humidified incubator containing 95% air and 5% CO_2_ at 37°C. Cells grow up to 80%‐90%, cell passage was performed as following: cells are quickly washed 1‐2 times with PBS. Then, 0.25% trypsin was added to digest the cells under a microscope, followed by 10% FBS medium to terminate the digestion, and centrifuged at 800‐1000 rpm for 5 minutes. The supernatant was discarded, the growth medium was added and resuspended and pipetted, and passaged at a ratio of 1 to 3. Experiments were initiated at passages 6‐8.

### 2.3 Cytotoxicity

We used a Cell Counting Kit‐8 (CCK8, CK04, Dojindo Kumamoto, Japan) to determine the cytotoxicity of TNF‐α and calycosin in C2C12 cells. Five thousand cells per well were seeded into 96‐well plates and incubated for 24 hours. Cells at 85% confluence were stimulated with different concentrations of TNF‐α (0, 20, 40, 60, 80 and 100 ng/mL), calycosin (0, 3.75, 7.5, 15, 30, 60,120 µg/mL) and AICAR (0, 0.5, 1, 2, 4, 8, 16 µmol/L) at 37°C for 24 hours. Fifteen microlitres of CCK8 was added to each well, and the samples were incubated at 37°C for 3 hours. Optical density (OD) was measured at 490 nm using a microplate reader (Multiskan™ FC, Thermo Fisher, USA).

### 2.4 Reverse‐transcription quantitative PCR (RT‐qPCR)

C2C12 cells (3 × 10^5^ cells/mL) were plated into 6‐well plates and grown to 70%‐80% confluence. Total RNA was extracted using TRIzol reagent (Takara, Tokyo, Japan) and reverse transcribed into cDNA using a Prime Script RT Reagent Kit (Takara) according to the manufacturer's instructions. RT‐qPCR was performed using a CFX96™ Real‐Time System (Bio‐Rad, Hercules, California, USA) using SYBR Green (SYBR Premix Ex Taq™ II; Takara) for fluorescence quantification. The following thermal program used for RT‐qPCR: predenaturation (95°C, 30 seconds), denaturation (95°C, 5 seconds, 35 cycles), annealing (55‐60°C, 30 seconds), extension (72°C, 1 minutes) and final extension (72°C, 10 minutes). Relative mRNA expression was calculated using the 2^−∆∆Ct^ method. The sequence of the primers as following:

CARM1 Forward: 5′‐TGCAGAACCACACGGACT‐3′; Reverse: 5′‐TACTTTGCCAGGGATGACCAC‐3′.

GAPDH Forward: 5′‐CATGGCCTTCCGTGTTCCTA‐3′; Reverse: 5′‐CCTGCTTCACCACCTTCTTG A‐3′.

### 2.5 Small interfering RNA and LC3B plasmid transfection

For CARM1‐small interfering (si)RNA transfection, C2C12 cells were seeded into 6‐well plates, grown to 75% confluence, then transfected with siCARM1 or scrambled siRNA (siCtrl) for 24‐48 hours using Lipofectamine 3000 (Invitrogen, Carlsbad, CA, USA) according to the manufacturer's instructions. The targeting sequence of siCARM1 is following:

CARM1‐1 (5′‐3′) Sense: GCAGACAGUCCUUCAUCAUTT; (5′‐3′) Antisense: AUGAUGAAUUACUGUCUGCCC;

CARM1‐2 (5′‐3′) Sense: GGACAAGAUCGUUCUAGAUTT; (5′‐3′) Antisense: AUCUAGAACGAUCUUGUCCTT;

CARM1‐3 (5′‐3′) Sense: CCCACUAUGCAGUCAACAATT; (5′‐3′) Antisense: UUGUUGACUGCAUAGUGGGCA.

To induce myotube atrophy, the C2C12 myotubes were treated with 40 ng/mL TNF‐α or phosphate‐buffered saline (PBS) for 24 hours following 24 hours of incubation with siCARM1. The expression of CARM1 was determined using immunoblotting and RT‐qPCR analyses.

For LC3A/B plasmid transfection in C2C12 myoblasts, cells were seeded into 12‐well plates and transfected with LC3B plasmids using Lipofectamine 3000 (Invitrogen, Carlsbad, CA, USA) according to the manufacturer's instructions. Images were captured using a fluorescence microscope (×200, XDP‐EC, MPS 30, Leica).

### 2.6 Histological and immunohistochemical analysis for TGF‐β

The kidneys were fixed in 4% formaldehyde at 4°C overnight and then embedded in paraffin. Tissue sections (4 μm) were deparaffinized in xylene and rehydrated in graded alcohol. Masson trichome staining and H&E staining were subjected as previously reported.^25^ Tubulointerstitial fibrosis was analysed by the percentage of fibrotic area in tubulointerstitial area using the Image pro plus software (Media Cybernetics, Rockville, MD, USA) in 200X randomly fields.

For immunohistochemical staining of TGF‐β, endogenous peroxidase was blocked with 3% H2O2 and non‐specific proteins were blocked with 10% goat serum or rabbit serum for 30 minutes. The sections were then incubated with indicated primary antibodies at 4°C for overnight, respectively, followed by incubation with an HRP‐conjugated secondary antibody at room temperature for 30 minutes. The sections were further counterstained with haematoxylin and then assessed under a microscope in a blinded manner by two pathologists.

### 2.7 Haematoxylin‐eosin staining (H&E)

Tibialis anterior muscle tissues were fixed in 4% paraformaldehyde for 48 hours and embedded in paraffin. The paraffin‐embedded samples were sliced into 6‐µm‐thick sections, then stained with haematoxylin‐eosin (H&E) and examined using a microscope (Nikon, Japan). For myofibre cross‐sectional area (CSA) analysis, six images were randomly collected for each sample and analysed using ImageJ software. At least 1500 myofibres per TA muscle were measured.

### 2.8 TUNEL apoptosis assay

Deparaffinized and rehydrated sections were treated with proteinase K for 30 minutes at 37°C, washed with PBS 3 times (5 minutes each time), then incubated with FITC‐labelled TUNEL staining buffer ( TdT/dUTP as 2/29) for 2 hours in incubator at 37°C. After washing with PBS again, converter‐POD was added to cover the tissue for 30 minutes. Tissues were washed with PBS and the stained with DAPI for 10 minutes. The TUNEL‐positive green cells were imaged using a fluorescence microscope (×200, Nikon, ECLIPSE TI‐SR, Japan).

### 2.9 Measurement of ROS

Intracellular ROS levels were evaluated using a 2′‐7′‐dichlorofluorescein diacetate (H_2_DCFDA) fluorescent probe (GeneCopoeia, C263, USA). C2C12 cells were seeded in triplicate at a density of 2 × 10^4^ cells/well in 24‐well plates, pre‐treated with calycosin (7.5 µg/mL) for 24 hours, then incubated with or without TNF‐α (40 ng/mL) for an additional 24 hours. Following treatment, H_2_DCFDA (10 μmol/L) was added to each well, and the cells were incubated in the dark at 37°C for 30 minutes. Finally, the cells were washed with cold PBS for 3 minutes and stained with DAPI for 10 minutes. Images were captured using a fluorescence microscope (×200).

Muscles and C2C12 cells were collected for analysis of oxidative stress. The activities of SOD, GSH‐Px, and CAT, and MDA levels were measured using detection kits (SOD: A00‐1; MDA: A003‐1; CAT: A007‐1; GSH‐Px: A005, Jiancheng Bioengineering Ltd., Nanjing, China) according to the manufacturer's instructions.

### 2.10 Hoechst 33342 staining

Hoechst 33342 fluorescence staining was used to evaluate cell apoptosis. Cells were plated in 6‐well plates and treated with 7.5 µg/mL calycosin for 24 hours, then incubated with or without TNF‐α (40 ng/mL) for an additional 24 hours. One millilitre of buffer, 5 µL of Hoechst 33342 and 5 µL of PI were added to the samples, and the cells were incubated at 4°C for 30 minutes, then rinsed with cold PBS for 3 minutes. The cells were immediately visualized using a fluorescence microscope (×200, XDP‐EC, MPS 30, Leica).

### 2.11 Transmission electron microscopy (TEM)

To evaluate autophagosome and mitochondrial changes, TA muscle was analysed using TEM. Fresh TA muscle was cut into 10 mm^3^ portions, then fixed in 0.5% glutaraldehyde, 2% paraformaldehyde, 7% saccharose, and 4% polyvinylpyrrolidone in 0.1 mol/L cacodylate buffer and post‐fixed in 2% osmic acid in 0.1 mol/L cacodylate buffer for 1 hour. The samples were dehydrated, embedded and sliced into 0.1‐μm‐thick sections. Images were captured using a microscope (Leica UC7 HT7700 HITACHI).

### 2.12 Immunocytochemical staining

Tibialis anterior muscles were collected and fixed with fresh 4% paraformaldehyde for 2 days and sectioned. The sections were deparaffinized and rehydrated, and endogenous peroxidase activity was blocked by incubating the samples with 3% H_2_O_2_ for 15 minutes in the dark. Antigen retrieval was performed by heating the samples in a pressure cooker in citrate buffer (Saiguo, Guangzhou, China) for 10 minutes. The samples were then incubated at room temperature for 40 minutes and washed three times with PBS. The samples were blocked using 5% BSA for 30 minutes at room temperature. The tissue sections were incubated with primary antibodies, including MuRF1 (1:200, Boster, USA), MAFbx (1:200, Affinity, USA), LC3A/B (1:200, Affinity), ATG7 (1:200, Affinity), CARM1 (1:200, Affinity) and H3R17me2a (1:200, Affinity) at 4°C overnight. The cells were washed with PBS three times, then incubated with a biotin‐conjugated secondary antibody for 40 minutes at 37°C. A 3,3'‐diaminobenzidine (DAB) colour development kit (Gene Tech, GK500705, Shanghai, China) was used according to the manufacturer's instructions. Finally, the cells were counterstained with haematoxylin and imaged using a microscope. Signal intensity was scored as follows: 0 (no staining), 1 (weak staining), 2 (moderate staining) and 3 (strong staining). The percentage of positively stained cells was divided into four categories: <25% (1), 25‐50% (2), 51‐75% (3) and > 76% (4). The final staining scores were calculated by multiplying the intensity score by the staining percentage. The range of possible scores was 0 to 12.

### 2.13 Immunofluorescence staining

Cells were seeded on slide covers in 24‐well plates, grown to 80% confluence, and treated as described in 2.10. Then, the cells were fixed with 4% paraformaldehyde for 15 minutes, washed with PBS three times (10 minutes each), permeabilized with 0.25%Triton X‐100 in 3% BSA for 10 minutes, washed with PBS three times, then blocked in 5% BSA at room temperature for 40 minutes. After washing with PBS, the cells were incubated with antibodies against LC3A/B (1:250, Affinity), AMPK (1:200, Affinity), SKP2 (1:200, Affinity), CARM1 (1:250, Affinity) and H3R17me3a (1:200, Affinity) at 4°C overnight. Then, the cells were washed with PBS and incubated with a fluorescent secondary antibody (DyLight 594, E032420, EarthOx, USA; DyLight 488, A23220, Abbkine) in the dark for 60 minutes, stained with DAPI for 10 minutes, then washed twice with PBS. Images were captured using a fluorescence microscope (Eclipse Ti‐S, Nikon, Japan).

### 2.14 Immunoblotting

C2C12 cells and gastrocnemius muscles were lysed in RIPA buffer, and protein concentrations were determined using a BCA protein assay kit (Thermo Fisher Scientific, USA). Equal amounts of protein were loaded onto 10% SDS polyacrylamide gels, subjected to electrophoresis (at constant pressure, 60 V for 30 minutes, 120 V for 60 minutes), transferred to PVDF membranes (Millipore, Billerica, MA, USA) under a constant current of 350 mA for 120 minutes, blocked with 5% BSA or 5% milk in 1× TBST for 1 hour, then incubated with the following primary antibodies at 4°C overnight: GAPDH (1:2000; EarthOx, USA), MuRF1 (1:1000, Boster, USA), MAFbx (1:1000, Affinity), Myogenin (1:1000, Affinity), LC3A/B (1:800, Affinity), ATG7 (1:800, Affinity), SKP2 (1:1000, Affinity), CARM1 (1:1000, Affinity), H3R17me3a (1:1000, Affinity), AMPK (1:1000, Affinity, AF6423), p‐AMPK (1:1000; Affinity, AF3424), FOXO3a (1:1000, Affinity, AF5418), and p‐FOXO3a (1:1000, Affinity). The membranes were washed with 1× TBST three times (10 minutes each) and incubated with species‐matched HRP‐conjugated secondary antibodies (1:5000, Affinity) for 1 hours, then washed with 1× TBST 3 times (10 minutes each). The blots were visualized with enhanced chemiluminescence (ECL) HRP substrate reagent (WBKLS0500, Millipore, USA) using a MiniChemi610 system (304002L, Beijing, China).

### 2.15 Statistical analysis

All statistical analyses were performed using SPSS 22.0 software (SPSS, Chicago, USA) and GraphPad Prism software 5.0 (CA, US). Data are presented as the mean ± SD and were analysed using one‐way ANOVA. Post hoc Bonferroni's test was performed when ANOVA showed significant differences among variables. *P*‐values < 0.05 were considered statistically significant.

## 3 RESULTS

### 3.1 Calycosin improved bodyweight and renal function and prevented histopathological changes in 5/6 Nx rats

**FIGURE 1:**
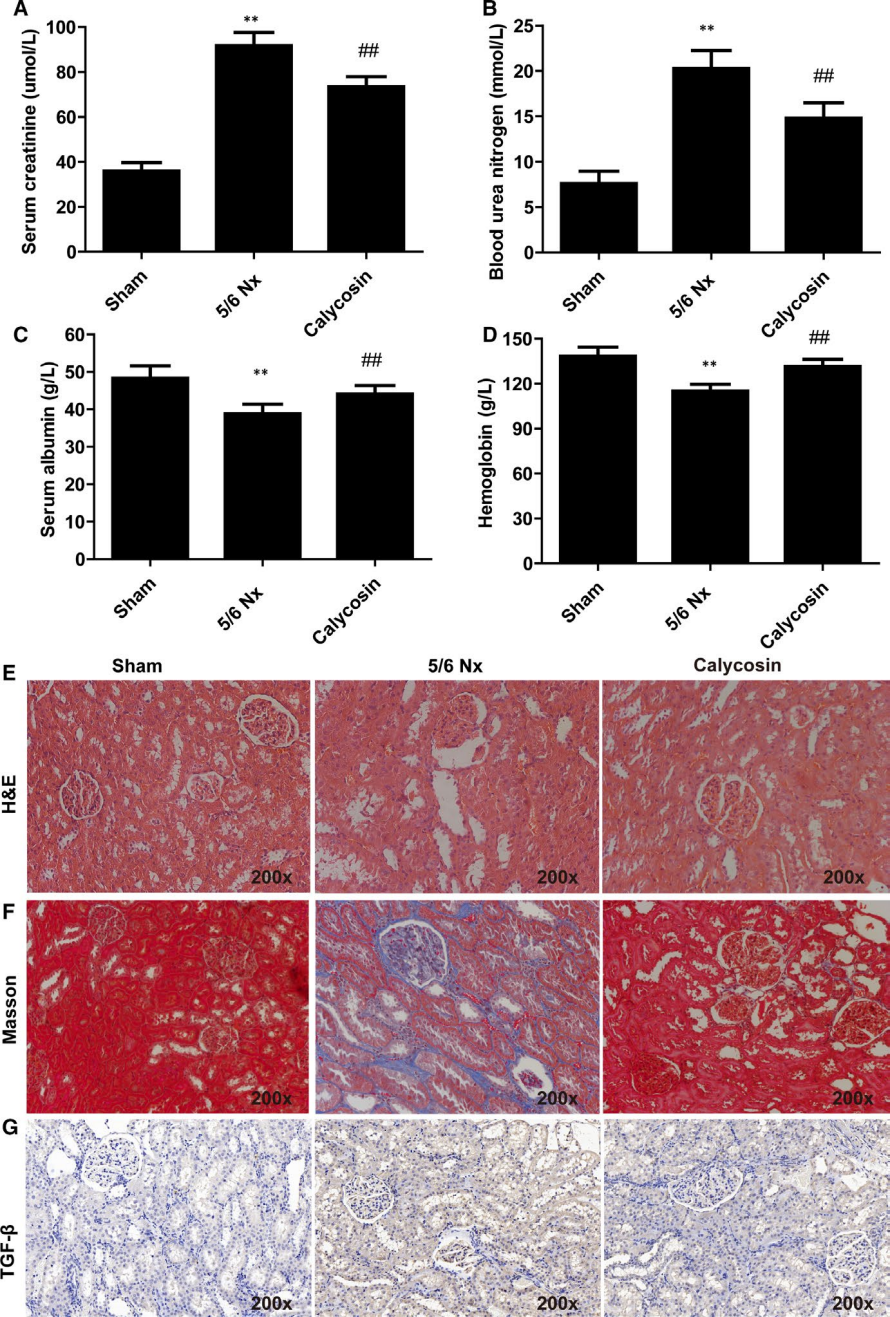
Effect of calycosin on renal histology and fibrosis in 5/6 Nx rats. The level of Scr (A), BUN (B), Alb (C), HB (D)in serum of rats in different group. Representative images of H&E (E), Masson trichrome (F) and TGF‐β (G) stained rat kidney sections (200×). *P < 0.05 compared to the sham group. ^##^ *P* < 0.01 compared to the 5/6 Nx model group

We evaluated the effects of calycosin on bodyweight and Scr, BUN, HB and Alb levels in 5/6 Nx rats. The results showed that the bodyweights in the 5/6 Nx group were significantly lower than those in the sham group from 2 weeks to 16 weeks after surgery (Table 1). Treatment with calycosin improved the bodyweights of 5/6 Nx rat after 4 weeks of treatment (Table 1). Levels of Scr, BUN, HB and Alb were significantly increased in 5/6 Nx rats 8 weeks after surgery compared with those in sham rats (Figure 1A‐D). Treatment with calycosin resulted in reduced levels of Scr, BUN, HB and Alb compared to those in the 5/6 Nx group (Figure 1A‐D).

**TABLE 1:** The total bodyweight of the rats in different group

Group (n = 10)	Time (weeks)					
	0	2	4	8	12	16
Sham	196.4 + 4.4	248.4 + 7.4	287.8 + 7.9	379.4 + 12.0	470.8 + 14.1	554.2 + 16.0
5/6 Nx	196.5 + 4.3	244.1 + 6.9	241.6 + 7.2**	285.2 + 9.2**	362.2 + 19.9**	438.9 + 16.3**
Calycosin	195.7 + 3.7	247.4 + 5.8	242.2 + 6.5**	290.3 + 9.0**	410.9 + 16.2**,##	498.0 + 13.1**,##

** *P* < 0.01 compared to sham group.

^##^ *P* < 0.01 compared to 5/6 Nx model group.

Renal fibrosis results from excessive accumulation of extracellular matrix and occur in every type of CKD.^26^ We performed histological analysis of kidney using H&E staining, Masson trichrome staining and immunohistochemical staining of TGF‐β (Figure 1E‐G). The kidney of sham rats showed normal morphology. In contrast, 5/6 Nx rats showed vacuolation and mild mesangial proliferation, with segmental sclerosis of remnant glomeruli, focal renal fibrosis, enlargement of the tubular lumen, tubular epithelial cell vacuolization, tubular atrophy, interstitial expansion and infiltration of inflammatory cells (Figure 1E‐G). In addition, 5/6 Nx rats had elevated kidney levels of TGF‐β compared with those in the sham group 8 weeks after surgery (Figure 1E‐G). Calycosin treatment significantly reduced the occurrence of renal lesions and inhibited renal fibrosis caused by the 5/6 Nx procedure (Figure 1E‐G). These results showed that calycosin improved bodyweight, improved renal function, and ameliorated histopathological changes in 5/6 Nx rats.

### 3.2 Calycosin alleviated muscle atrophy in 5/6 Nx rats

**FIGURE 2:**
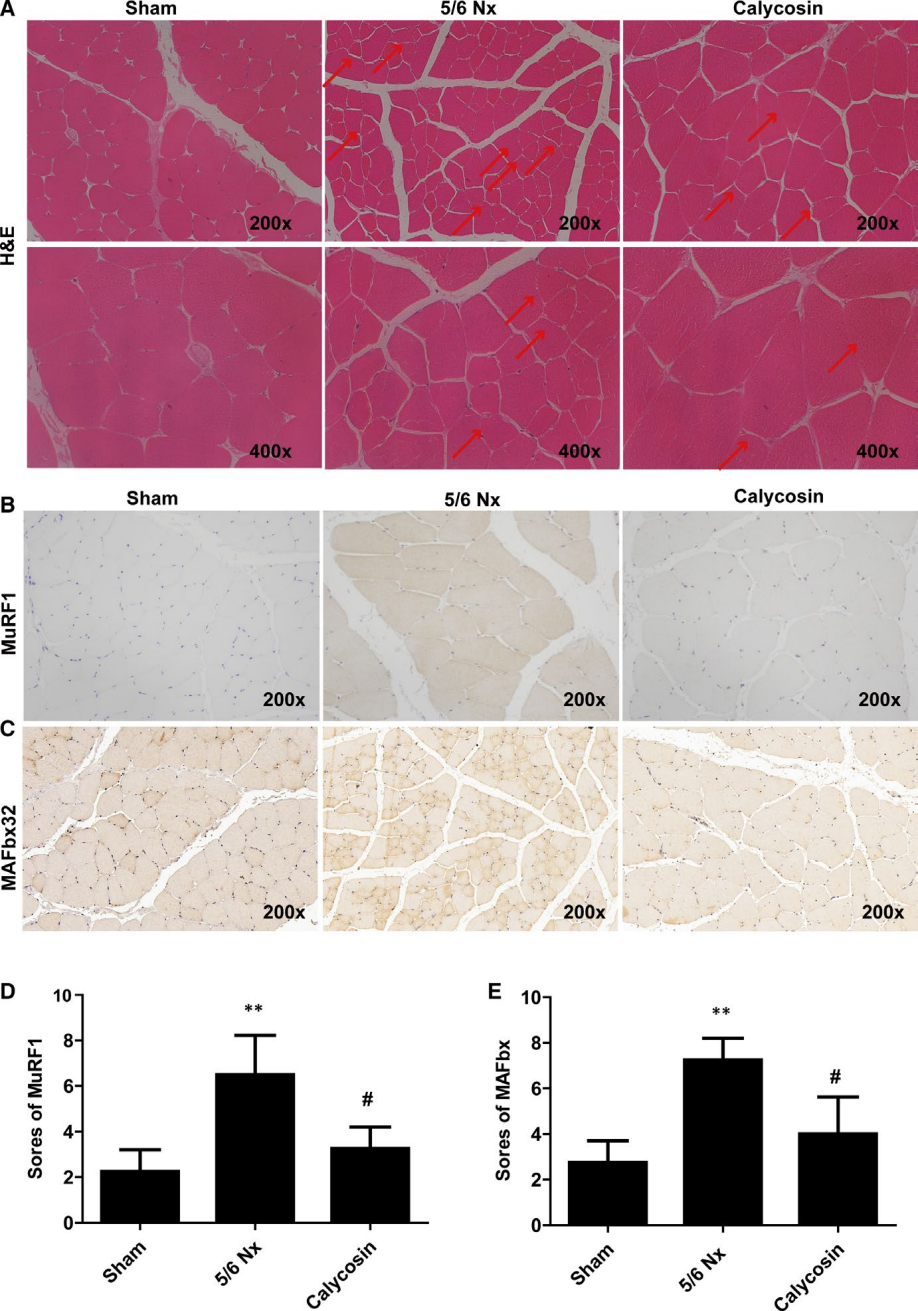
Effect of calycosin on muscle histology, apoptosis, and MuRF1 and MAFbx expression levels in muscle of 5/6 Nx rats. A, Representative images of H&E stained rat muscles (200x). B‐E, Representative images and scores of IHC staining of MuRF1 and MAFbx in rat muscles (×200). Bar = 50 µm. *P < 0.05 compared to the sham group. ^##^ *P* < 0.01 compared to the 5/6 Nx model group

Histomorphological analysis using H&E staining of TA muscles was performed. The result showed that TA muscle fibre cross‐sectional areas were decreased in 5/6 Nx rats (Figure 2A), and these decreases were reversed by treatment with calycosin (Figure 2A).

Muscle‐atrophy F‐Box (MAFbx)/atrogin‐1 and muscle ring‐finger‐1 (MuRF‐1) are important indicators of muscle atrophy. We evaluated the expression of MAFbx and MuRF‐1 using IHC staining and immunoblotting. The results showed that the expression levels of MAFbx and MuRF‐1 were increased in TA and GAS muscles of 5/6 Nx rats (Figure 2B‐D), and these increases were inhibited by administration of calycosin (Figure 2B‐D). These results showed that calycosin may inhibit skeletal muscle atrophy in 5/6 Nx rats.

### 3.3 calycosin inhibited autophagy and oxidative stress in skeletal muscle of 5/6 Nx rats

**FIGURE 3:**
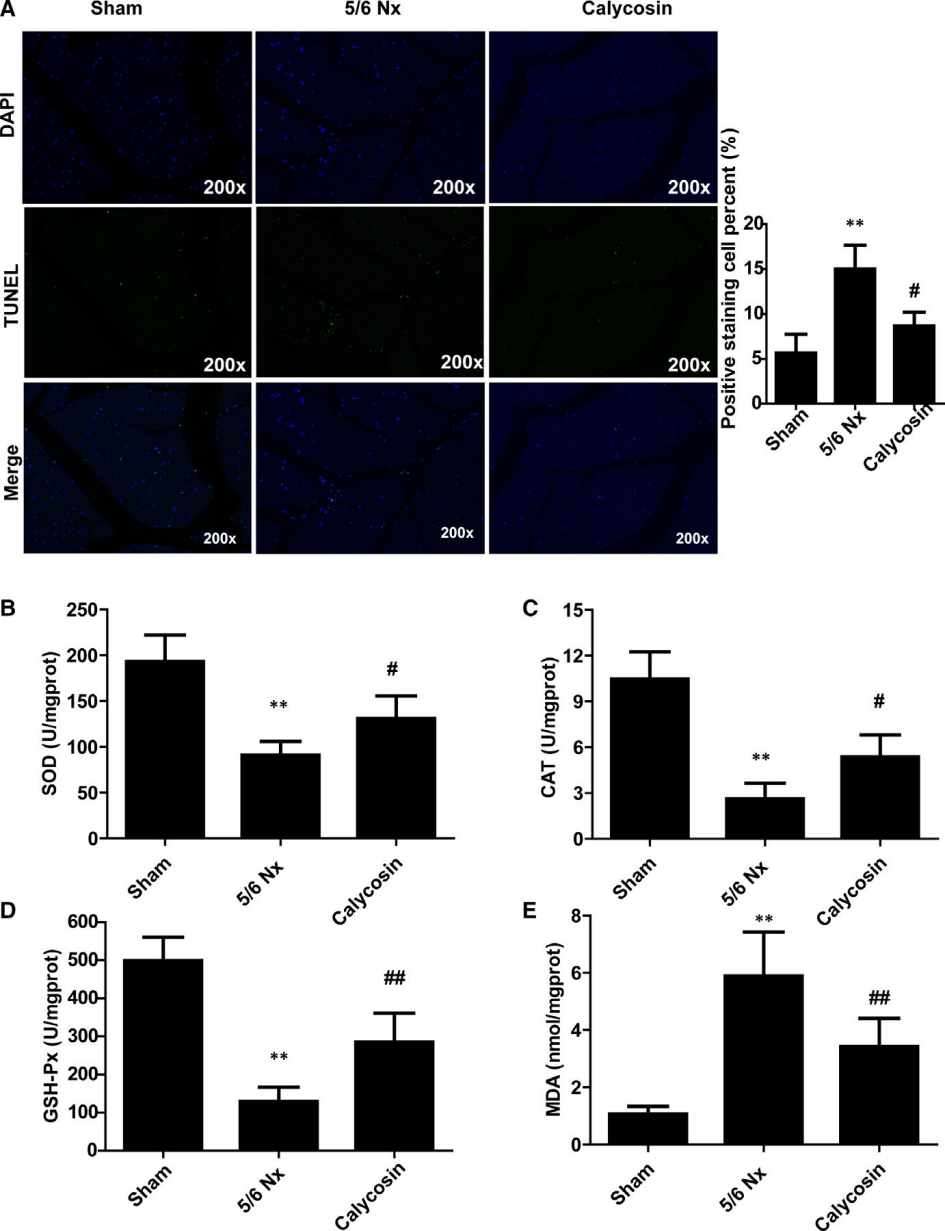
Effect of calycosin on cell apoptosis and oxidative stress in muscle of 5/6 Nx rats. A, Representative images of TUNEL in TA muscle of rat for evaluation of apoptosis (×200). B‐E, The activity of SOD, CAT, and GSH‐Px and MDA levels, in rat muscle. *P < 0.05 compared to the sham group. ^##^ *P* < 0.01 compared to the 5/6 Nx model group

Next, TUNEL staining was performed to access cell apoptosis, the results showed more intense positive staining in 5/6 Nx rats, which indicated increased cell apoptosis (Figure 3A). Treatment with calycosin resulted in decreased TUNEL staining intensity compared to that in the 5/6 Nx group (Figure 3A).

Moreover, to evaluate oxidative stress, we measured the activities of SOD, CAT, and GSH‐Px and quantified MDA in GAS muscles. The activities of SOD, CAT and GSH‐Px were lower, and MDA levels were higher, in the muscle of 5/6 Nx rats than those in the muscle of sham rats (Figure 3B‐E). Treatment with calycosin blocked 5/6 Nx‐induced oxidative stress (Figure 3B‐E), which indicated that calycosin exerted antioxidant effects.

**FIGURE 4:**
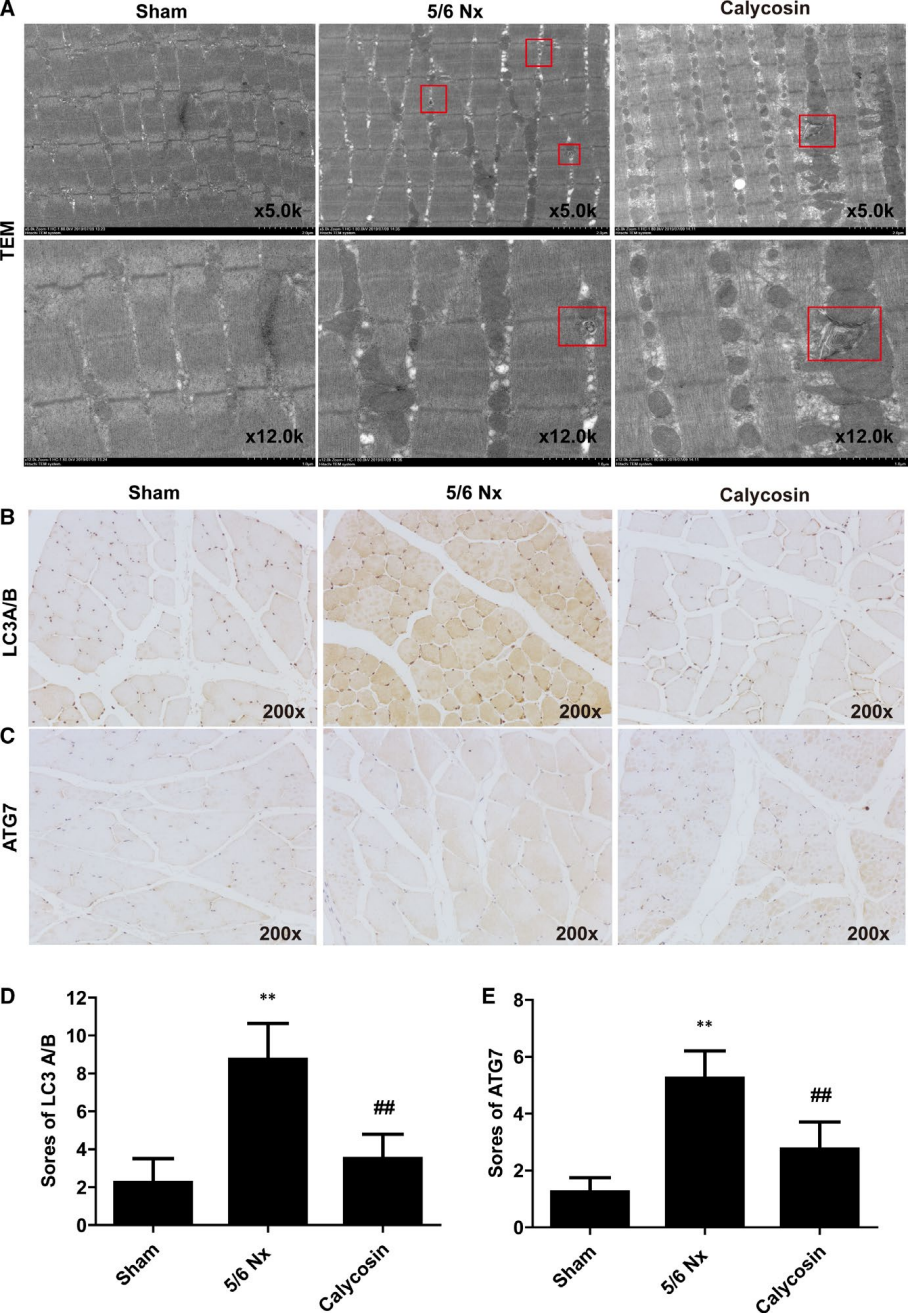
Effect of calycosin on autophagy in muscle of 5/6 Nx rats. A, Representative images of autophagosomes observed in muscle of rat using TEM. B‐E, Representative images and scores of LC3A/B and ATG7 expression in muscle of rats using IHC staining (×200). Bar = 50 µm. *P < 0.05 compared to the sham group. ^##^ *P* < 0.01 compared to the 5/6 Nx model group

We also performed TEM analysis to evaluate autophagosome formation and mitochondrial morphology in muscle atrophy in 5/6 Nx rats. In the sham group muscles, autophagosomes were not abundant, and the mitochondrial crests were dense and neat. But in the muscle of model rats, the mitochondrial matrix electron density decreased, ridges moved around, shortened and decreased. Besides, mitochondrial volume increased, swelling and vacuolation occurred, mitochondrial ridges and membranes were lost, and mitochondrial breakage increased (Figure 4A). Treatment with calycosin reduced the number of autophagosomes compared to that in the 5/6 Nx group (Figure 4A), which suggested that calycosin inhibited autophagy in CKD induced muscle atrophy. In addition, the expression levels of LC3A/B and ATG7 in TA muscles were evaluated using IHC staining. The results showed that LC3A/B and ATG7 staining intensities were stronger in the muscle of 5/6 Nx rats than those in sham rats (Figure 4B‐E). Treatment with calycosin resulted in lower levels of LC3A/B and ATG7 than those in 5/6 Nx rats (Figure 4B‐E). These results suggested that calycosin protected against 5/6 Nx‐induced autophagy and muscle atrophy.

### 3.4 Calycosin down‐regulated the expression of AMPK/SKP2/CARM1/H3R17me2a in skeletal muscle of 5/6 Nx

**FIGURE 5:**
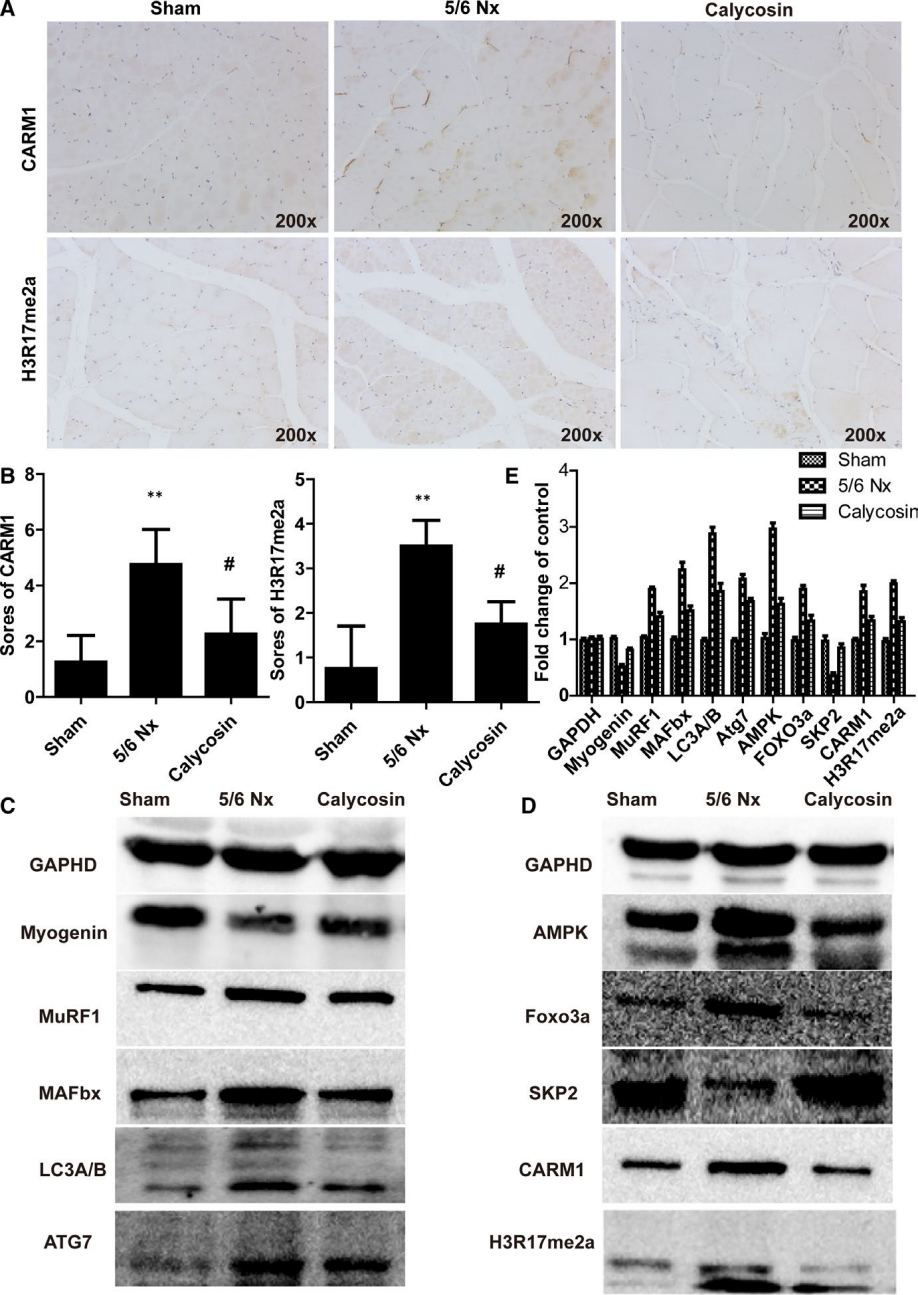
Effect of calycosin on the expression of CARM1 and H3R17me2a, and related proteins in rat muscles. A‐B, Representative images and scores of the expression of CARM1 and H3R17me2a in muscle sections using IHC staining (×200). Bar = 50 µm. *P < 0.05 compared to the sham group. ^##^ *P* < 0.01 compared to the 5/6 Nx model group. C‐E, Representative immune blots of key proteins involved in muscle atrophy and proteins associated with the AMPK/SKP2/CARM1 signalling pathway

We quantified the levels of proteins in the AMPK/SKP2/CARM1/H3R17me2a signalling pathway, which is involved in autophagy and oxidative stress. The expression levels of CARM1 and H3R17me2a were higher in the muscle of 5/6 Nx rats than those in muscle of sham rats (Figure 5A‐B). Treatment with calycosin prevented 5/6 Nx‐induced increases in the expression of CARM1 and H3R17me2a (Figure 5A‐B).

We also evaluated key proteins involved in muscle atrophy using immunoblot analysis. The results showed that the levels of MAFbx, MuRF‐1, FOXO3a, LC3A/B, ATG7, AMPK, CARM1 and H3R17me2a were up‐regulated, and the levels of SKP2 and p‐FOXO3a were down‐regulated in muscle of 5/6 Nx rats (Figure 5C‐D). Treatment with calycosin blocked these 5/6 Nx‐induced changes (Figure 5C‐D). These results showed that calycosin reduced 5/6 Nx‐induced autophagy and oxidative stress in skeletal muscle through regulation of the AMPK/SKP2/CARM1/H3R17me2a signalling pathway.

### 3.5 si‐CARM1 transfection ameliorated oxidative stress and autophagy induced myotube atrophy in vitro

**FIGURE 6:**
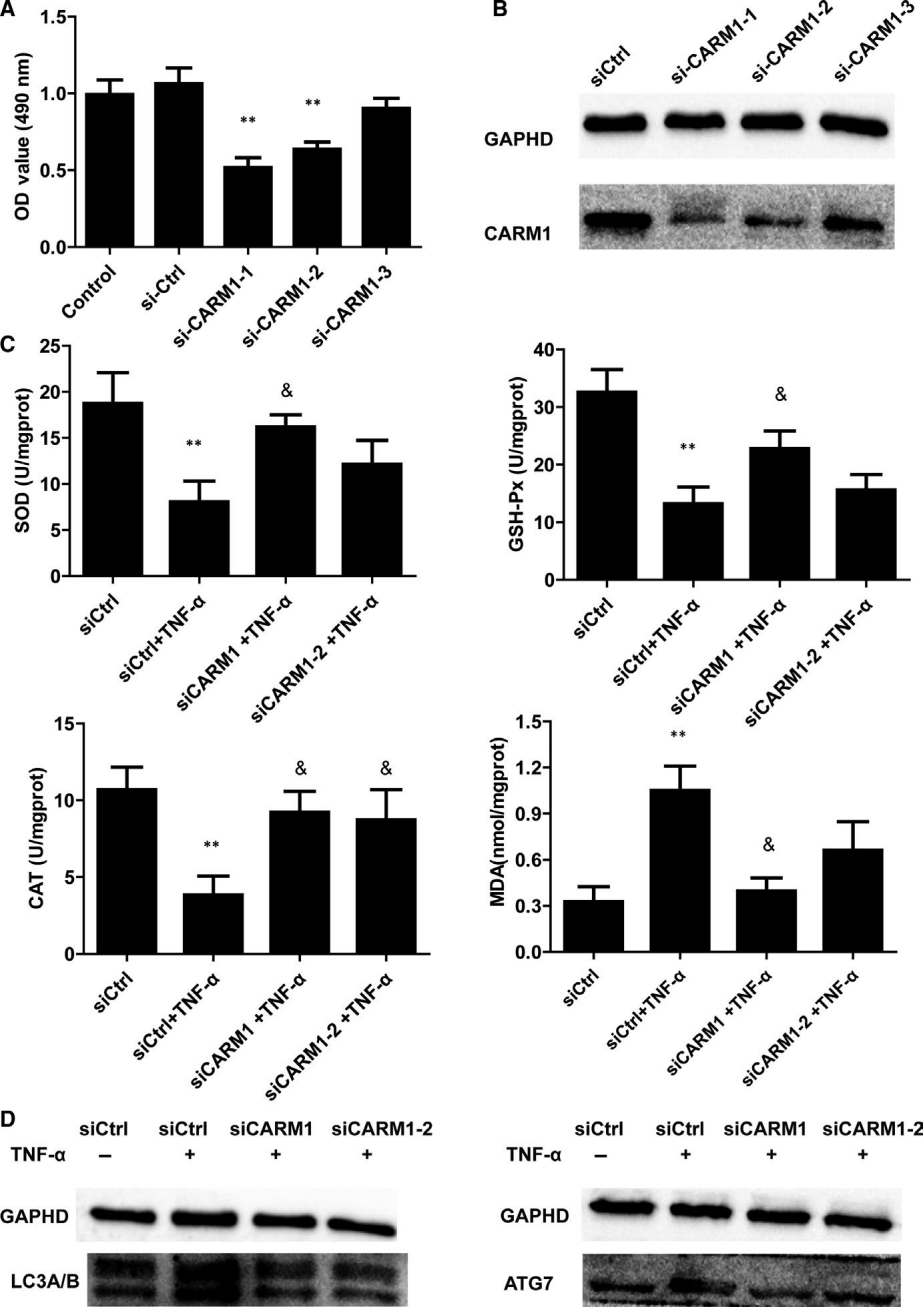
Effect of CARM1 siRNA transfection in C2C12 cells in vitro. A‐B, Relative expression of CARM1 qRT‐PCR and Western blot with siRNA transfection in C2C12 cells. *P < 0.05 compared to siCtrl. C, The levels of SOD, CAT, GSH‐Px and apoptosis in C2C12 cells with siRNA control or siRNA CARM1 transfection. *P < 0.05 compared to control siRNA. ^&^ *P* < 0.05 compared to control siRNA + TNF‐α. D, Representative protein expression of LC3A/B and ATG7 by Western blot in C2C12 cells with siCtrl or siRNA CARM1 transfection

Then, we examined the role of CARM1 in myotube atrophy. siRNA of CARM1 and siRNA of control were transfected in C2C12 for 48 hours and then cells were treated with TNF‐α for 24 hours. Results of immunoblotting and RT‐qPCR analyses confirmed the expression of CARM1 was decreased after transfection (Figure 6A‐B). Besides, results showed the level of SOD, CAT, GSH‐Px were increased, and MDA level decreased after siRNA CARM1 transfection, compared to siCtrl (Figure 6C). In addition, siRNA CARM1 transfection deceased LC3A/B and ATG7 expression in TNF‐α‐induced C2C12 cells (Figure 6D).

### 3.6 Calycosin ameliorated myotube atrophy in TNF‐α‐induced C2C12 cells

**FIGURE 7:**
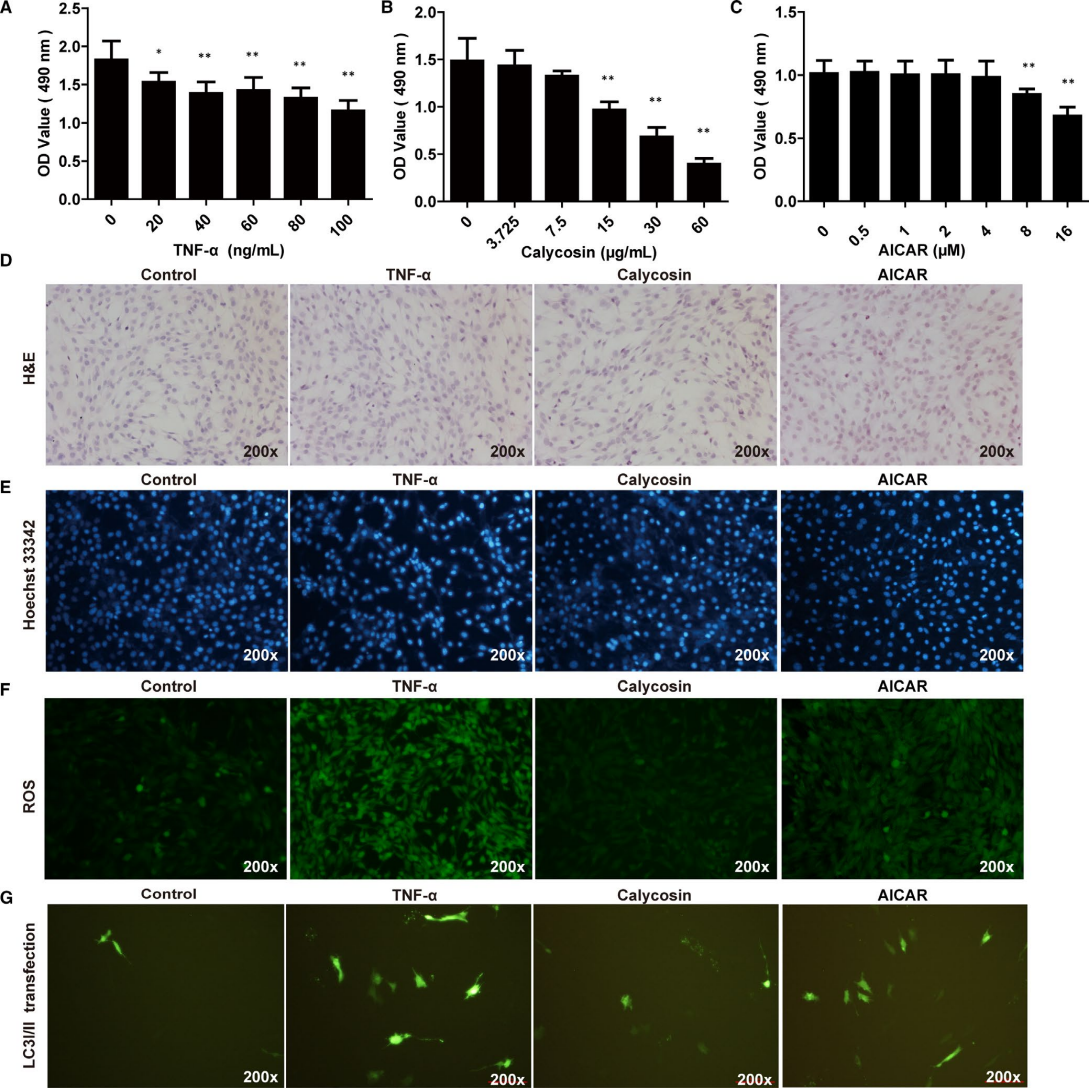
Effect of calycosin on myotube atrophy in TNF‐α induced C2C12 cells in vitro. A‐C, Cytotoxicity of TNF‐α, calycosin and AICAR in C2C12 cells. *P < 0.05 compared to control cells. D, Representative images of H&E staining of C2C12 cells treated with TNF‐α or calycosin (×200). *P < 0.05 compared to control cells. ^##^ *P* < 0.01 compared to TNF‐α induced cells. E, Representative images of cell apoptosis in C2C12 cells treated with TNF‐α or calycosin using Hoechst 33342 staining. *P < 0.05 compared to control cells. ^##^ *P* < 0.01 compared to TNF‐α‐induced cells. F, Representative images of ROS production in C2C12 cells treated with TNF‐α or calycosin. G, Representative images of LC3A/B plasmid transfection in C2C12 cells treated with TNF‐α or calycosin

We evaluated the effects of calycosin on TNF‐α‐induced C2C12 cells. The results showed that treatment with 40 ng/mL TNF‐α reduced cell viability (Figure 7A), and calycosin at 3.75, 7.5 µg/mL did not induce cytotoxicity (Figure 7B), so we used 7.5 µg/mL for further in vitro experiments. AICAR at 0.5, 1, 2, 4 µmol/L did not induce cytotoxicity, so 4 µmol/L was used for cell recovery experiments (Figure 7C). For haematoxylin and eosin staining, 20‐30 myotube cross‐section areas in each group were randomly calculated by image J pro plus software to evaluate myotube atrophy. The results showed that 40 ng/mL TNF‐α induced myotube atrophy in C2C12 cells, and 7.5 µg/mL calycosin reduced TNF‐α‐induced myotube atrophy, and AICAR inhibited the effect of calycosin (Figure 7C).

We also evaluated apoptosis in C2C12 cells using Hoechst 33342 staining. The results showed that TNF‐α induced apoptosis in C2C12 cells (Figure 7D), and 7.5 µg/mL calycosin inhibited TNF‐α‐induced apoptosis, which was reversed by AICAR (Figure 7D). These results showed that calycosin ameliorated myotube atrophy and apoptosis in TNF‐α‐induced C2C12 cells.

### 3.7 Calycosin decreased autophagy and oxidative stress in TNF‐α‐induced C2C12 cells

We evaluated ROS production and LC3A/B plasmid transfection in C2C12 cells. The results showed that TNF‐α promoted ROS production in C2C12 cells, and calycosin decreased TNF‐α‐induced ROS production, (Figure 7E). In addition, TNF‐α treatment resulted in increased LC3A/B plasmid transfection efficiency in C2C12 cells (Figure 7F), and calycosin decreased TNF‐α‐induced LC3A/B plasmid transfection efficiency (Figure 7F). But, AICAR also increased the ROS production and LC3A/B plasmid transfection efficiency in cells with calycosin treatment. These results showed that calycosin decreased autophagy and oxidative stress in TNF‐α induced C2C12 cells, and the effect was partially abrogated by AMPK activator (AICAR).

### 3.8 Calycosin improved myotube atrophy in TNF‐α‐induced C2C12 cells by regulating the AMPK/SKP2/CARM1 signalling pathway

**FIGURE 8:**
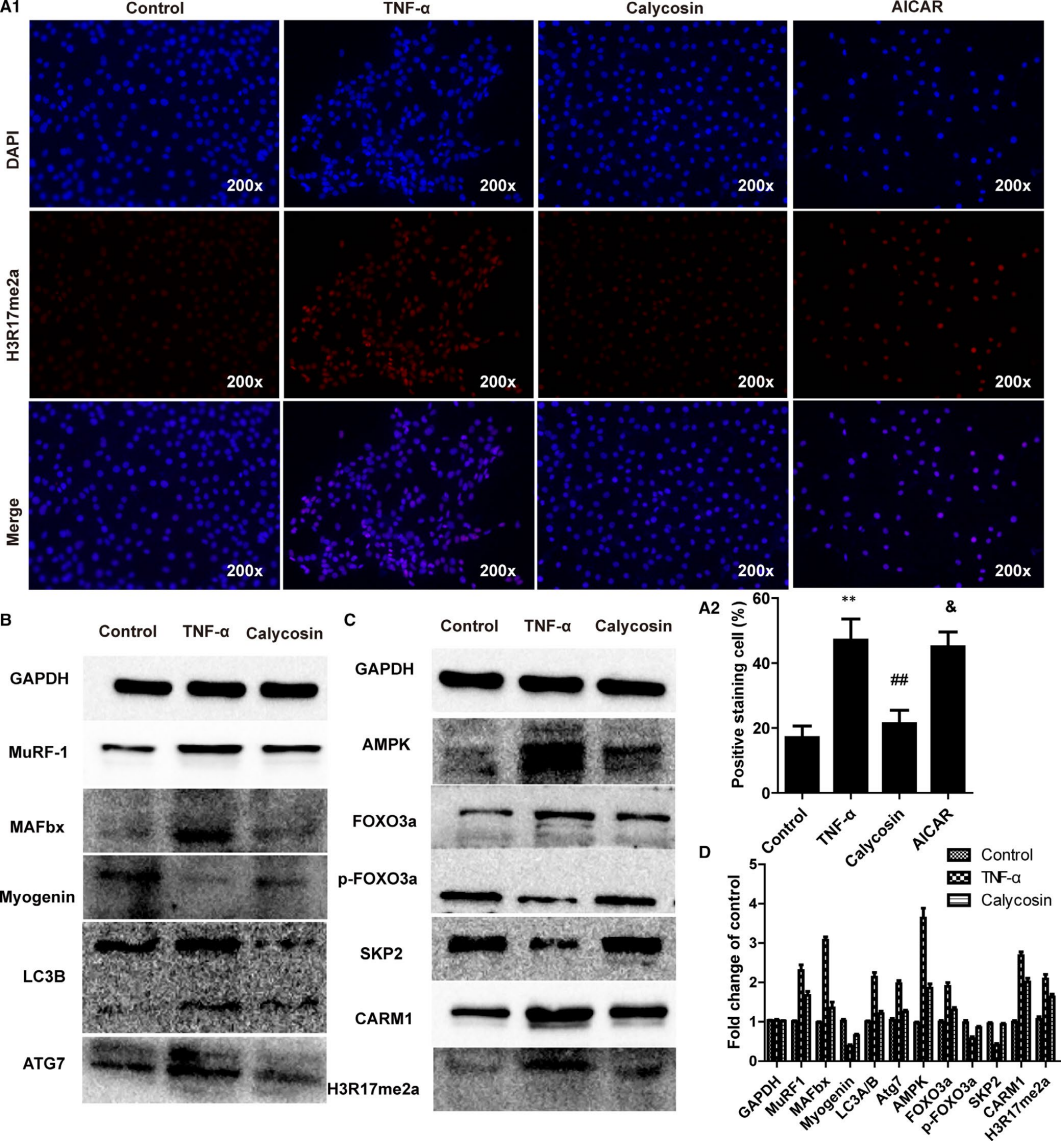
Effect of calycosin on the expression of proteins associated with the AMPK/SKP2/CARM1 signalling pathway in TNF‐α‐induced C2C12 cells in vitro. A, Representative images of H3R17me2a in C2C12 cells treated with TNF‐α or calycosin. B‐C, Representative immunoblot of key proteins associated with the AMPK/SKP2/CARM1 signalling pathway in C2C12 cells treated with TNF‐α or calycosin. D, Semiquantitative analysis of the immunoblot bands

We examined the effects of calycosin on the expression of CARM1 and H3R17me2a, and components of the AMPK/SKP2/CARM1 signalling pathway, in C2C12 cells. The results showed that TNF‐α up‐regulated the expression of CARM1 and H3R17me2a in C2C12 cells, as evidenced by immunofluorescence staining (Figure 8A). Calycosin abrogated TNF‐α‐induced up‐regulation of CARM1 and H3R17me2a in C2C12 cells (Figure 8A). Moreover, immunoblotting results showed that the protein levels of MAFbx, MuRF‐1, FOXO3a, LC3A/B, ATG7, AMPK, CARM1 and H3R17me2a were up‐regulated, and the levels of SKP2 and p‐FOXO3a were down‐regulated, in TNF‐α‐induced C2C12 cells (Figure 8B‐C). These changes were prevented by calycosin treatment (Figure 8B‐C). These results suggested that calycosin alleviated myotube atrophy by regulating the AMPK/SKP2/CARM1 signalling pathway in TNF‐α‐induced C2C12 cells.

## 4 DISCUSSION

Skeletal muscle atrophy is associated with pathological changes such as increased inflammation, mitochondrial dysfunction, elevated apoptosis, reduced protein synthesis and increased protein degradation. These processes are driven by oxidative stress, autophagy and ubiquitin‐proteasome pathway activation.^27^ Therefore, therapeutic strategies that target these mechanisms have great potential for prevention of CKD‐induced skeletal muscle atrophy. Calycosin has been extensively studied. A previous study showed that calycosin exerted anti‐autophagy, anti‐apoptosis, and anti‐inflammatory effects against focal cerebral ischemia and reperfusion injury in rats.^28^ Another study showed that calycosin inhibited oxidative stress and apoptosis through activation of ERa/b and enhanced Akt phosphorylation in cardiomyocytes.^29^ In addition, calycosin has been shown to ameliorate diabetes‐induced renal inflammation via the NF‐κB pathway in vitro and in vivo.^30^ However, the effects of calycosin on renal function and skeletal muscle atrophy in CKD have not been characterized. In this study, we showed that calycosin inhibited autophagy and oxidative stress in CKD‐induced skeletal muscle atrophy in rats and in TNF‐α induced myotube atrophy in C2C12 cells through regulation of the AMPK/SKP2/CARM1 signalling pathway.

Recent studies showed that localized accumulation of ROS could activate autophagic pathways and induce muscle atrophy.^31^ Excessive production of ROS is mitigated by antioxidant enzymes, such as SOD, and CAT, and GSH‐PX.^32^ Superoxide dismutase can be classified as two distinct species: Cu/ZnSOD is present in the cytoplasm and Mn‐SOD is present in mitochondria.^33^ A previous study showed that CKD‐induced skeletal muscle atrophy resulted in decreased levels of mitochondrial SOD and SOD1, slightly reduced mitochondrial catalase activity, and increased levels of mitochondrial H_2_O_2_, ROS, and malondialdehyde (MDA).^34^ In this study, we obtained similar results and also found that GSH‐Px activity was decreased in 5/6 Nx rats. In addition, calycosin administration improved mitochondrial oxidative capacity in the muscle tissue of CKD rats.

Studies have shown that FOXO3a, a member of the FOXO family of proteins, may play a role in initiation of protein degradation during muscle atrophy,^35^ resulting in reduced ROS generation through transcriptional activation of SOD and CAT.^36^ In addition, oxidative stress has been reported to activate AMP‐activated protein kinase (AMPK),^37^ resulting in myotube atrophy through activation of the ubiquitin‐mediated protein proteasomal degradation pathway involving muscle ring‐finger‐1 (MuRF‐1) and muscle atrophy F‐Box (MAFbx).^38^, ^39^, ^40^ Levels of MuRF1 and/or MAFbx have been shown to be up‐regulated in skeletal muscles,^41^ under multiple conditions such as starvation, ageing, diabetes, cancer^42^ and muscle disuse induced by renal failure in animals and humans.^43^ We showed that the ubiquitin ligases MuRF1, MAFbx and FOXO3a were activated and apoptosis was increased in skeletal muscle of 5/6 Nx rats, and calycosin treatment abrogated these increases, which indicated that calycosin may protect against muscle atrophy.

Autophagy is an evolutionary self‐preservation process that cleans out damaged cells and protein aggregates to regenerate new healthier cells. However, up‐regulation of autophagy can induce apoptosis.^44^ The primary process in the autophagy‐related signalling pathway is associated with isolation membrane formation,^45^ which lead to a mature autophagosome through autophagy‐related genes (ATG) protein recruitment,^46^ specifically such as Atg7, Atg12, Atg4 and microtubule‐associated protein 1 light chain 3 (LC3).^47^ Over‐activation of autophagy in skeletal muscles has been shown to result in protein degradation and muscle loss independently or cooperatively during denervation, disuse, fasting or oxidative stress.^10^ LC3 and the autophagy adaptor protein p62/SQSTM1 (p62) are key indicators of autophagosome formation and targeting of mitochondria for lysosomal degradation.^48^ In skeletal muscle, FOXO is key players in controlling LC3, Beclin 1 and p62.^49^ In our study, Atg7, LC3,^50^and FOXO3a protein expression levels were significantly increased in the muscle of 5/6 Nx rats and in TNF‐α induced C2C12 cells compared with those in the corresponding control groups. These effects were blocked by treatment with calycosin. In addition, the LC3II/LC3I ratio was increased in the muscle of 5/6 Nx rat and in TNF‐α induced C2C12 cells compared with that in the corresponding control groups.

CARM1 is a co‐activator for transcription factors and plays crucial role in regulating muscle fibre type, oxidative metabolism and myogenesis.^51^ A recent study showed that CARM1 played a role in muscular atrophy. Increased CARM1 expression is positively related to muscle mass loss in denervation induced mice. Interestingly, CARM1 knockdown resulted in an autophagic deficit in myofibres, which halted muscle atrophy process and reduced the protein level of MuRF1 and MAFbx both in vivo and in vitro through FoxO3‐dependent transcription.^19^ In addition, CARM1 has been shown to exhibit methyltransferase activity, resulting in methylation of CREB‐binding protein (CBP) and histone H3 at arginine 17/26/42.^52^, ^53^ Furthermore, CARM1 has been shown to exert effect on autophagy‐related and lysosomal genes through regulating transcription of S phase kinase‐associated protein 2 (SKP2), which is responsible for CARM1 degradation. The role of AMPK in metabolism and autophagy has been well characterized. A study showed that decreased nuclear AMPK‐FoxO3 activity caused increased expression of SKP2‐E3 ubiquitin ligase, which resulted in decrease CARM1 levels and autophagy in aged hearts.^54^ However, under nutrient (glucose) starvation conditions, AMPK‐dependent phosphorylation of FOXO3a was increased in the nucleus, which transcriptionally repressed SKP2, and resulted in increased levels of CARM1,histone H3 Arg17 dimethylation (H3R17me2), and other autophagy‐related gene activity.^18^

The 5‐amino‐4‐imidazole carboxamide ribonucleoside (AICAR) has been used to activate AMPK in various tissues of the body, including skeletal muscle. After administration, it was converted to ZMP (AICAR monophosphate), an AMP mimic that activates AMPK without altering intracellular adenine nucleotide levels. Studies have shown that treatment of C2C12 myotubes with AICAR increased expression of FOXO1, FoxO3a, Atrogin‐1, MuRF‐1 and two other FoxO target genes (LC3 and Bnip3), inducing degradation/ decomposition of proteins.^55^ These effects are independent of Akt/ mTOR. In addition, AMPK activator promotes autophagy in skeletal muscle probably by inhibits mTORC1 activity and promoting autophagy flux. On the one hand, AMPK directly phosphorylates ULK1 at multiple sites and targets autophagy‐related protein 9 (Atg9) and beclin‐1.^56^ On the other hand, AMPK activates FoxO3a, inhibits SKP2, promotes CARM1 and H3R17me2a levels, and activates transcription and expression of autophagy‐related genes. In our study, we showed that increased autophagy in CKD‐induced skeletal muscle atrophy was accompanied by activated AMPK and increased level of FOXO3a, decreased SKP2 levels, and increased CARM1 and H3R17me2 levels. These changes were prevented by calycosin administration in vivo and in vitro. AICAR partially reversed the positive effect of calycosin on autophagy and oxidative stress in C2C12 cells. Furthermore, our study showed that CARM1 may be a crucial regulator of skeletal muscle atrophy and suggested that calycosin may be a potential therapy for skeletal muscle atrophy induced by CKD, malnutrition or other conditions.

Previous study showed that calycosin suppresses the expression of pro‐inflammatory cytokines via p62/Nrf2‐linked HO‐1 induction in rheumatoid arthritis synovial fibroblasts.^57^ Another study showed that calycosin treatment decreased the levels of TNF‐α, interleukin (IL)‐6, IL‐1β, and MDA in acute pancreatitis (AP) mice and Alzheimer's Disease mouse.^58^, ^59^ Additionally, calycosin significantly reduced cerulein‐induced pancreatic oedema inhibited myeloperoxidase (MPO) activity and increased SOD activity via the p38 MAPK and NF‐κB signal pathways.^58^ However, in our study, we treated C2C12 cells with TNF‐α in in vitro experiment which is a proinflammatory cytokine. Could anti‐inflammatory effect of calycosin be upstream of its anti‐oxidative and anti‐autophagy effects in this experiment? This is a limitation that we did not investigate whether the anti‐inflammatory effect of calycosin could be upstream of its anti‐oxidative and anti‐autophagy effects. Further study should be performed in our next experiment to get the results.

## 5 CONCLUSIONS

Our results showed that anti‐oxidative enzyme (SOD, CAT and GSH‐Px) activity was decreased, and ROS and MDA productions were increased in CKD‐induced muscle atrophy, and in TNF‐α‐induced C2C12 cells. In addition, the expression of MuRF1, MAFbx and FOXO3a, and autophagy markers such as ATG7 and LC3A/B, was increased, and AMPK/SKP2/CARM1 pathway activity was altered in CKD‐induced muscle atrophy and TNF‐α‐induced C2C12 cells. These results suggested that these changes may promote myotube atrophy through activation of proteasomal degradation, oxidative stress and autophagy. Our study identified calycosin as a potential therapeutic agent to prevent skeletal muscle atrophy through reduced oxidative stress and autophagy via regulation of AMPK/SKP2/CARM1 signalling pathway.

## CONFLICT OF INTEREST

The authors declare that they have no competing interests to disclose.

## AUTHOR CONTRIBUTION

**Lianbo Wei:** Project administration (lead); Supervision (lead). **Rong Hu:** Investigation (lead); Methodology (lead); Writing‐original draft (lead); Writing‐review & editing (lead). **Ming‐qing Wang:** Data curation (equal); Investigation (supporting); Software (supporting). **Ling‐yu Liu:** Data curation (supporting); Methodology (supporting); Validation (supporting). **Hai‐yan You:** Conceptualization (supporting); Investigation (supporting); Methodology (supporting). **Xiao‐hui Wu:** Conceptualization (supporting); Formal analysis (supporting); Methodology (supporting). **Yang‐yang Liu:** Resources (supporting); Visualization (supporting). **Yan‐jing Wang:** Funding acquisition (supporting); Resources (supporting). **Lu Lu:** Data curation (supporting); Formal analysis (lead); Visualization (supporting). **Wei Xiao:** Funding acquisition (equal); Supervision (supporting).

## References

[1] Levin A , Tonelli M , Bonventre J , et al. Global kidney health 2017 and beyond: a roadmap for closing gaps in care, research, and policy. Lancet. 2017;390(10105):1888‐1917. 28434650 10.1016/S0140-6736(17)30788-2

[2] Matsushita K , van der Velde M , Astor BC , et al. Association of estimated glomerular filtration rate and albuminuria with all‐cause and cardiovascular mortality in general population cohorts: a collaborative meta‐analysis. Lancet. 2010;375(9731):2073‐2081. 20483451 10.1016/S0140-6736(10)60674-5 PMC3993088

[3] Carrero JJ , Stenvinkel P , Cuppari L , et al. Etiology of the protein‐energy wasting syndrome in chronic kidney disease: a consensus statement from the International Society of Renal Nutrition and Metabolism (ISRNM). J Ren Nutr. 2013;23(2):77‐90. 23428357 10.1053/j.jrn.2013.01.001

[4] Schiaffino S , Dyar KA , Ciciliot S , Blaauw B , Sandri M . Mechanisms regulating skeletal muscle growth and atrophy. FEBS J. 2013;280(17):4294‐4314. 23517348 10.1111/febs.12253

[5] Robinson KA , Baker LA , Graham‐Brown M , Watson EL . Skeletal muscle wasting in chronic kidney disease: the emerging role of microRNAs. Nephrol Dial Transplant. 2019; 10.1093/ndt/gfz193 31603229

[6] Wang XH , Mitch WE . Mechanisms of muscle wasting in chronic kidney disease. Nat Rev Nephrol. 2014;10(9):504‐516. 24981816 10.1038/nrneph.2014.112 PMC4269363

[7] Schardong J , Marcolino M , Plentz R . Muscle atrophy in chronic kidney disease. Adv Exp Med Biol. 2018;1088:393‐412. 30390262 10.1007/978-981-13-1435-3_18

[8] Anderson LJ , Liu H , Garcia JM . Sex differences in muscle wasting. Adv Exp Med Biol. 2017;1043:153‐197. 29224095 10.1007/978-3-319-70178-3_9

[9] Savikj M , Kostovski E , Lundell LS , Iversen PO , Massart J , Widegren U . Altered oxidative stress and antioxidant defence in skeletal muscle during the first year following spinal cord injury. Physiol Rep. 2019;7(16):e14218. 31456346 10.14814/phy2.14218 PMC6712236

[10] Bloemberg D , Quadrilatero J . Autophagy, apoptosis, and mitochondria: molecular integration and physiological relevance in skeletal muscle. Am J Physiol Cell Physiol. 2019;317(1):C111‐C130. 31017800 10.1152/ajpcell.00261.2018 PMC6689753

[11] Powers SK , Smuder AJ , Judge AR . Oxidative stress and disuse muscle atrophy: cause or consequence? Curr Opin Clin Nutr Metab Care. 2012;15(3):240‐245. 22466926 10.1097/MCO.0b013e328352b4c2 PMC3893113

[12] Matsui Y . Pathological state or cause of sarcopenia. Clin Calcium. 2017;27(1):45‐52. 28017945

[13] Kaur N , Gupta P , Saini V , et al. Cinnamaldehyde regulates H2 O 2 ‐induced skeletal muscle atrophy by ameliorating the proteolytic and antioxidant defense systems. J Cell Physiol. 2019;234(5):6194‐6208. 30317570 10.1002/jcp.27348

[14] Powers SK , Ji LL , Kavazis AN , Jackson MJ . Reactive oxygen species: impact on skeletal muscle. Comp Physiol. 2011;1(2):941‐969. 10.1002/cphy.c100054 PMC3893116 23737208

[15] Masiero E , Agatea L , Mammucari C , et al. Autophagy is required to maintain muscle mass. Cell Metab. 2009;10(6):507‐515. 19945408 10.1016/j.cmet.2009.10.008

[16] Mizushima N , Komatsu M . Autophagy: renovation of cells and tissues. Cell. 2011;147(4):728‐741. 22078875 10.1016/j.cell.2011.10.026

[17] Zhang YY , Gu LJ , Huang J , et al. CKD autophagy activation and skeletal muscle atrophy‐a preliminary study of mitophagy and inflammation. Eur J Clin Nutr. 2019;73(6):950‐960. 30607007 10.1038/s41430-018-0381-x

[18] Shin HJ , Kim H , Oh S , et al. AMPK‐SKP2‐CARM1 signalling cascade in transcriptional regulation of autophagy. Nature. 2016;534(7608):553‐557. 27309807 10.1038/nature18014 PMC5568428

[19] Liu Y , Li J , Shang Y , Guo Y , Li Z . CARM1 contributes to skeletal muscle wasting by mediating FoxO3 activity and promoting myofiber autophagy. Exp Cell Res. 2019;374(1):198‐209. 30500392 10.1016/j.yexcr.2018.11.024

[20] Zhang Y , Shi S , Guo J , You Q , Feng D . On‐line surface plasmon resonance‐high performance liquid chromatography‐tandem mass spectrometry for analysis of human serum albumin binders from Radix Astragali. J Chromatogr A. 2013;1293:92‐99. 23639128 10.1016/j.chroma.2013.04.015

[21] Xiao W , Han L , Shi B . Isolation and purification of flavonoid glucosides from Radix Astragali by high‐speed counter‐current chromatography. J Chromatogr B Analyt Technol Biomed Life Sci. 2009;877(8–9):697‐702. 10.1016/j.jchromb.2009.01.034 19213616

[22] Gao J , Liu ZJ , Chen T , Zhao D . Pharmaceutical properties of calycosin, the major bioactive isoflavonoid in the dry root extract of Radix astragali. Pharm Biol. 2014;52(9):1217‐1222. 24635389 10.3109/13880209.2013.879188

[23] El‐Kott AF , Al‐Kahtani MA , Shati AA . Calycosin induces apoptosis in adenocarcinoma HT29 cells by inducing cytotoxic autophagy mediated by SIRT1/AMPK‐induced inhibition of Akt/mTOR. Clin Exp Pharmacol Physiol. 2019;46(10):944‐954. 31276230 10.1111/1440-1681.13133

[24] Ghosh SS , Massey HD , Krieg R , et al. Curcumin ameliorates renal failure in 5/6 nephrectomized rats: role of inflammation. Am J Physiol Renal Physiol. 2009;296(5):F1146‐F1157. 19225048 10.1152/ajprenal.90732.2008

[25] Zhang M , Guo Y , Fu H , et al. Chop deficiency prevents UUO‐induced renal fibrosis by attenuating fibrotic signals originated from Hmgb1/TLR4/NFkappaB/IL‐1beta signaling. Cell Death Dis. 2015;6:e1847. 26247732 10.1038/cddis.2015.206 PMC4558499

[26] Zhang D , Sun L , Xian W , et al. Low‐dose paclitaxel ameliorates renal fibrosis in rat UUO model by inhibition of TGF‐beta/Smad activity. Lab Invest. 2010;90(3):436‐447. 20142807 10.1038/labinvest.2009.149

[27] Bandyopadhaya A , Tzika AA , Rahme LG . Pseudomonas aeruginosa quorum sensing molecule alters skeletal muscle protein homeostasis by perturbing the antioxidant defense system. MBio. 2019;10(5):e02211‐19. 10.1128/mBio.02211-19 PMC6775459 31575771

[28] Wang Y , Ren Q , Zhang X , Lu H , Chen J . Neuroprotective mechanisms of calycosin against focal cerebral ischemia and reperfusion injury in rats. Cell Physiol Biochem. 2018;45(2):537‐546. 29402799 10.1159/000487031

[29] Liu B , Zhang J , Liu W , et al. Calycosin inhibits oxidative stress‐induced cardiomyocyte apoptosis via activating estrogen receptor‐alpha/beta. Bioorg Med Chem Lett. 2016;26(1):181‐185. 26620254 10.1016/j.bmcl.2015.11.005

[30] Zhang YY , Tan RZ , Zhang XQ , Yu Y , Yu C . Calycosin ameliorates diabetes‐induced renal inflammation via the NF‐kappaB pathway in vitro and in vivo. Med Sci Monit. 2019;25:1671‐1678. 30830898 10.12659/MSM.915242 PMC6413560

[31] Aucello M , Dobrowolny G , Musaro A . Localized accumulation of oxidative stress causes muscle atrophy through activation of an autophagic pathway. Autophagy. 2009;5(4):527‐529. 19221466 10.4161/auto.5.4.7962

[32] Ott M , Gogvadze V , Orrenius S , Zhivotovsky B . Mitochondria, oxidative stress and cell death. Apoptosis. 2007;12(5):913‐922. 17453160 10.1007/s10495-007-0756-2

[33] Zelko IN , Mariani TJ , Folz RJ . Superoxide dismutase multigene family: a comparison of the CuZn‐SOD (SOD1), Mn‐SOD (SOD2), and EC‐SOD (SOD3) gene structures, evolution, and expression. Free Radic Biol Med. 2002;33(3):337‐349. 12126755 10.1016/s0891-5849(02)00905-x

[34] Wang D , Wei L , Yang Y , Liu H . Dietary supplementation with ketoacids protects against CKD‐induced oxidative damage and mitochondrial dysfunction in skeletal muscle of 5/6 nephrectomised rats. Skelet Muscle. 2018;8(1):18. 29855350 10.1186/s13395-018-0164-z PMC5984473

[35] Sandri M , Sandri C , Gilbert A , et al. Foxo transcription factors induce the atrophy‐related ubiquitin ligase atrogin‐1 and cause skeletal muscle atrophy. Cell. 2004;117(3):399‐412. 15109499 10.1016/s0092-8674(04)00400-3 PMC3619734

[36] Huang C , Lin Y , Su H , Ye D . Forsythiaside protects against hydrogen peroxide‐induced oxidative stress and apoptosis in PC12 cell. Neurochem Res. 2015;40(1):27‐35. 25344274 10.1007/s11064-014-1461-5

[37] Auciello FR , Ross FA , Ikematsu N , Hardie DG . Oxidative stress activates AMPK in cultured cells primarily by increasing cellular AMP and/or ADP. Febs Lett. 2014;588(18):3361‐3366. 25084564 10.1016/j.febslet.2014.07.025 PMC4158911

[38] Shang F , Taylor A . Ubiquitin‐proteasome pathway and cellular responses to oxidative stress. Free Radic Biol Med. 2011;51(1):5‐16. 21530648 10.1016/j.freeradbiomed.2011.03.031 PMC3109097

[39] Ferraro E , Giammarioli AM , Chiandotto S , Spoletini I , Rosano G . Exercise‐induced skeletal muscle remodeling and metabolic adaptation: redox signaling and role of autophagy. Antioxid Redox Signal. 2014;21(1):154‐176. 24450966 10.1089/ars.2013.5773 PMC4048572

[40] Bodine SC , Baehr LM . Skeletal muscle atrophy and the E3 ubiquitin ligases MuRF1 and MAFbx/atrogin‐1. Am J Physiol Endocrinol Metab. 2014;307(6):E469‐E484. 25096180 10.1152/ajpendo.00204.2014 PMC4166716

[41] Foletta VC , White LJ , Larsen AE , Leger B , Russell AP . The role and regulation of MAFbx/atrogin‐1 and MuRF1 in skeletal muscle atrophy. Pflugers Arch. 2011;461(3):325‐335. 21221630 10.1007/s00424-010-0919-9

[42] Plant PJ , Brooks D , Faughnan M , et al. Cellular markers of muscle atrophy in chronic obstructive pulmonary disease. Am J Respir Cell Mol Biol. 2010;42(4):461‐471. 19520920 10.1165/rcmb.2008-0382OC

[43] Aniort J , Stella A , Philipponnet C , et al. Muscle wasting in patients with end‐stage renal disease or early‐stage lung cancer: common mechanisms at work. J Cachexia Sarcopenia Muscle. 2019;10(2):323‐337. 30697967 10.1002/jcsm.12376 PMC6463476

[44] Ravikumar B , Sarkar S , Davies JE , et al. Regulation of mammalian autophagy in physiology and pathophysiology. Physiol Rev. 2010;90(4):1383‐1435. 20959619 10.1152/physrev.00030.2009

[45] He C , Klionsky DJ . Regulation mechanisms and signaling pathways of autophagy. Annu Rev Genet. 2009;43:67‐93. 19653858 10.1146/annurev-genet-102808-114910 PMC2831538

[46] Hiensch AE , Bolam KA , Mijwel S , et al. Doxorubicin‐induced skeletal muscle atrophy: elucidating the underlying molecular pathways. Acta Physiol (Oxf). 2020;229(2):e13400. 10.1111/apha.13400 PMC7317437 31600860

[47] Smuder AJ , Kavazis AN , Min K , Powers SK . Exercise protects against doxorubicin‐induced markers of autophagy signaling in skeletal muscle. J Appl Physiol. 2011;111(4):1190‐1198. 21778418 10.1152/japplphysiol.00429.2011

[48] Palikaras K , Tavernarakis N . Mitochondrial homeostasis: the interplay between mitophagy and mitochondrial biogenesis. Exp Gerontol. 2014;56:182‐188. 24486129 10.1016/j.exger.2014.01.021

[49] Mammucari C , Milan G , Romanello V , et al. FoxO3 controls autophagy in skeletal muscle in vivo. Cell Metab. 2007;6(6):458‐471. 18054315 10.1016/j.cmet.2007.11.001

[50] Zhai J , Tao L , Zhang S , et al. Calycosin ameliorates doxorubicin‐induced cardiotoxicity by suppressing oxidative stress and inflammation via the sirtuin 1‐NOD‐like receptor protein 3 pathway. Phytother Res. 2020;34(3):649‐659. 31858651 10.1002/ptr.6557

[51] Stouth DW , Vanlieshout TL , Shen NY , Ljubicic V . Regulation of skeletal muscle plasticity by protein arginine methyltransferases and their potential roles in neuromuscular disorders. Front Physiol. 2017;8:870. 29163212 10.3389/fphys.2017.00870 PMC5674940

[52] Yi P , Wang Z , Feng Q , et al. Structural and functional impacts of ER coactivator sequential recruitment. Mol Cell. 2017;67(5):733‐743.e4. 28844863 10.1016/j.molcel.2017.07.026 PMC5657569

[53] Daujat S , Bauer UM , Shah V , Turner B , Berger S , Kouzarides T . Crosstalk between CARM1 methylation and CBP acetylation on histone H3. Curr Biol. 2002;12(24):2090‐2097. 12498683 10.1016/s0960-9822(02)01387-8

[54] Li C , Yu LU , Xue H , et al. Nuclear AMPK regulated CARM1 stabilization impacts autophagy in aged heart. Biochem Biophys Res Commun. 2017;486(2):398‐405. 28315332 10.1016/j.bbrc.2017.03.053

[55] Nakashima K , Yakabe Y . AMPK activation stimulates myofibrillar protein degradation and expression of atrophy‐related ubiquitin ligases by increasing FOXO transcription factors in C2C12 myotubes. Biosci Biotechnol Biochem. 2007;71(7):1650‐1656. 17617726 10.1271/bbb.70057

[56] Martin‐Rincon M , Morales‐Alamo D , Calbet J . Exercise‐mediated modulation of autophagy in skeletal muscle. Scand J Med Sci Sports. 2018;28(3):772‐781. 28685860 10.1111/sms.12945

[57] Su X , Huang Q , Chen J , et al. Calycosin suppresses expression of pro‐inflammatory cytokines via the activation of p62/Nrf2‐linked heme oxygenase 1 in rheumatoid arthritis synovial fibroblasts. Pharmacol Res. 2016;113(Pt A):695‐704. 27678042 10.1016/j.phrs.2016.09.031

[58] Ma R , Yuan F , Wang S , Liu Y , Fan T , Wang F . Calycosin alleviates cerulein‐induced acute pancreatitis by inhibiting the inflammatory response and oxidative stress via the p38 MAPK and NF‐kappaB signal pathways in mice. Biomed Pharmacother. 2018;105:599‐605. 29890468 10.1016/j.biopha.2018.05.080

[59] Song L , Li X , Bai XX , Gao J , Wang CY . Calycosin improves cognitive function in a transgenic mouse model of Alzheimer's disease by activating the protein kinase C pathway. Neural Regen Res. 2017;12(11):1870‐1876. 29239334 10.4103/1673-5374.219049 PMC5745842

